# T181. ABNORMAL FRONTAL AND PARIETAL SYNAPTIC GAIN RESPONSES IN FIRST EPISODE SCHIZOPHRENIA DURING A P300 TARGET DETECTION TASK

**DOI:** 10.1093/schbul/sby016.457

**Published:** 2018-04-01

**Authors:** Alvaro Diez, Alba Lubeiro Juárez, Benjamín Cañas, Aldara Alvarez-Astorga, Angel Nevado, Vicente Molina

**Affiliations:** 1 Universidad de Valladolid; 2 Hospital Clínico Universitario de Valladolid; 3 Universidad Complutense de Madrid

## Abstract

**Background:**

The “dysconnection hypothesis” proposes that schizophrenia is best understood in terms of aberrant brain functional integration and synaptic neuromodulation, which may underlie illness psychopathology and cognitive decline. Impairments in the P300 potential are well documented in schizophrenia and progressive over the years with the illness. We used Dynamic Causal Modeling (DCM) to investigate intrinsic (self-) connectivity in a frontoparietal cortical hierarchy during a P300 task; that is, how evoked activity results from the dynamics of coupled neural populations and how neural coupling changes with the experimental factors.

**Methods:**

Thirty-one patients with schizophrenia (16 first episode and 15 chronic patients) and 31 healthy controls underwent EEG recordings during an auditory oddball paradigm to elicit the P300 response. We studied 16 frontoparietal models (primary auditory, superior parietal, and superior frontal sources) and identified an optimal model of neural coupling, explaining illness ‘diagnosis’ and ‘chronicity’ effects, as well as their interactions with ‘task condition’.

**Results:**

The winning model included changes in connectivity in all 3 hierarchical levels. Compared to healthy controls, all patients (chronic and first episode) showed decreased self-inhibition – i.e., increased cortical excitability – in right superior parietal gyrus across task conditions. On the other hand, first episode patients – but not chronic – showed in the left frontal and parietal source a reversal of the normal synaptic gain changes in response to targets, relative to standard tones.

**Discussion:**

We confirmed that both subjects with chronic and first episode schizophrenia show a context-independent loss of parietal synaptic gain control. Importantly, in the highest levels of the hierarchy, first episode patients showed a specific abnormal gain modulation pattern in response to task-relevant stimuli not present in those chronically treated. Abnormal synaptic gain is plausibly caused by NMDA-receptor and/or GABAergic pathologies that change the excitability of superficial pyramidal cells, and may be independent of illness advance and chronic pharmacological treatment.

